# Supplementary material for: Evaluation of Enterotoxins and Antimicrobial Resistance in Microorganisms Isolated from Raw Sheep Milk and Cheese: Ensuring the Microbiological Safety of These Products in Southern Brazil
Source: Microorganisms. 2023 Jun 20;11(6):1618. doi: 10.3390/microorganisms11061618 (PMC10304779; doi:10.3390/microorganisms11061618)
Supplement: Supplementary file 1 [file microorganisms-11-01618-s001.zip › microorganisms-2418389-supplementary.pdf]

Supplementary table S1. Compilated results of raw sheep's milk microbiological quality and resistance studies.

Samples	Mesophiles	Total coliforms	*E. coli*	*S. aureus*	Enterotoxins	Nº of isolates	Identified species	Antimicrobial resistance	Resistance genes
**Farm 1 (milk)**	7.0E+3<br>8.7E+4<br>7.6E+4<br>6.1E+4<br>9.0E+4	3.1E+1<br>9.0E+0<br>1.7E+1<br>2.5E+1<br>2.6E+1	2.1E+1<br>0.0E+0<br>0.0E+0<br>2.0E+0<br>0.0E+0	2.8E+3<br>2.0E+3<br>2.7E+3<br>2.0E+3<br>1.8E+3	Negative<br>Negative<br>Negative<br>Negative<br>Negative	13	*S. simulans, S. sciuri*	Oxacillin, Cefovecin, Benzylpenicillin, Ceftiofur, Kanamycin, Neomycin, Pradofloxacin, Clindamycin, Tetracycline, Chloramphenicol, Trimethoprim, Erythromycin	*tet*L, *sul*1, *erm*B, *tet*M, *str*A
**Farm 2 (milk)**	3.8E+4<br>5.2E+4<br>4.8E+4<br>6.5E+4<br>3.2E+4	1.8E+3<br>3.7E+1<br>1.7E+2<br>6.1E+3<br>7.9E+3	3.0E+0<br>0.0E+0<br>0.0E+0<br>2.0E+0<br>9.0E+0	1.0E+3<br>5.3E+3<br>3.8E+3<br>1.8E+3<br>1.9E+3	Negative<br>Positive<br>Negative<br>Negative<br>Negative	1	*S. sciuri*	Oxacillin, Cefovecin	*erm*B, *tet*M
**Farm 3 (milk)**	6.0E+4<br>3.0E+4<br>3.3E+4<br>3.2E+4<br>3.3E+4	4.0E+2<br>8.7E+2<br>4.5E+3<br>3.8E+2<br>4.1E+3	1.9E+2<br>7.0E+1<br>1.9E+2<br>1.3E+2<br>1.3E+2	2.2E+3<br>4.0E+3<br>4.6E+3<br>4.0E+3<br>2.4E+3	Negative<br>Negative<br>Negative<br>Negative<br>Negative	10	*S. simulan, S. sciuri, S. aureus*	Oxacillin, Cefovecin, Benzylpenicillin, Ceftiofur, Kanamycin, Neomycin, Pradofloxacin, Clindamycin, Tetracycline, Chloramphenicol, Trimethoprim, Doxycycline, Enrofloxacin, Amoxicillin, Marbofloxacin, Cephalotin, Ampicillin, Rifanpicin	*tet* L, *sul*1, *sul*2, *erm*B, *tet*M, *str*A

Supplementary table S2. Compilated results of raw sheep's cheese microbiological quality and resistance studies.

Samples	Mesophiles	Total coliforms	*E. coli*	*S. aureus*	Enterotoxins	Nº of isolates	Identified species	Antimicrobial resistance	Resistance genes
**Feta-Type**	4.4E+5	2.6E+2	0.0E+0	0.0E+0	Negative	8	*S. lentus*,*S. sciuri*	Rifanpicin, Benzylpenicillin, Oxacillin, Cefovecine, Ceftiofur, Clindamycin	*tet*L, *sul*1,*erm*B, *tet*M, *str*A
	4.5E+5	0.0E+0	0.0E+0	0.0E+0	Negative				
	1.4E+5	0.0E+0	0.0E+0	0.0E+0	Negative				
	5.4E+6	1.9E+2	0.0E+0	0.0E+0	Negative				
	6.0E+6	1.5E+5	0.0E+0	0.0E+0	Negative				
**Colonial**	8.3E+3	0.0E+0	0.0E+0	0.0E+0	Negative	3	*S. pseudintermedius*, *S. chromogenes*	Benzylpenicillin, Oxacillin, Cephalothin, Cefovecine, Ceftiofur, Pradofloxacin, Erythromycin, Clindamycin, Tetracycline	*erm*B, *tet*M, *AAC*(6), *str*A
	3.0E+4	0.0E+0	0.0E+0	0.0E+0	Negative				
	3.4 E+4	0.0E+0	0.0E+0	0.0E+0	Negative				
	1.3E+5	0.0E+0	0.0E+0	0.0E+0	Negative				
	2.9E+5	0.0E+0	0.0E+0	0.0E+0	Negative				
**Fresh**	1.7E+5	1.2E+3	0.0E+0	6.4E+1	Negative	0	Not performed	Not performed	Not performed
	3.5E+5	2.4E+3	0.0E+0	1.8E+1	Negative				
	3.1E+5	8.1E+2	0.0E+0	1.0E+1	Negative				
	3.0E+5	1.3E+3	2.6E+2	6.0E+1	Negative				
	4.7E+5	3.2E+3	9.3E+2	0.0E+0	Negative				
**Pecorino-Type**	2.1E+5	0.0E+0	0.0E+0	0.0E+0	Negative	4	*S. lentus*, *S. warneri*	Rifanpicin, Benzylpenicillin, Erythromycin	*sul*1, *tet*W, *str*A
	2.0E+5	0.0E+0	0.0E+0	0.0E+0	Negative				
	4.1E+6	1.1E+3	0.0E+0	0.0E+0	Negative				
	6.4E+6	2.8E+3	0.0E+0	0.0E+0	Negative				
	2.7E+5	1.2E+2	0.0E+0	1.5E+2	Negative				
